# Translation and Population-Based Validation of the Arabic Version of the Fullerton Advanced Balance Scale

**DOI:** 10.3390/healthcare14020187

**Published:** 2026-01-12

**Authors:** Fayaz Khan

**Affiliations:** Department of Physical Therapy, Faculty of Medical Rehabilitation Sciences, King Abdulaziz University, Jeddah 22252, Saudi Arabia; fayazrkhan@gmail.com

**Keywords:** Fullerton Advanced Balance Scale, balance, fall, translation, validation, reliability

## Abstract

**Background/Objective:** This study aimed to translate the Fullerton Advanced Balance Scale (FAB) into Arabic and assess the instrument’s reliability and validity. **Methods:** The study was carried out in two distinct stages: (i) the translation and adaptation process utilizing the ‘forward-back’ translation method and (ii) the psychometric evaluation of the Arabic version of the FAB-A among a sample of 68 older persons residing in the community. **Results:** The internal consistency of the FAB-A was excellent (Cronbach’s alpha = 0.86). The Intraclass Correlation Coefficient (ICC) for the inter-rater tests (ICC = 0.96, *p* ≤ 0.001) and the intra-rater tests (ICC = 0.95, *p* ≤ 0.001) were excellent and significant. The scale showed a strong correlation with the Berg Balance Scale (r = 0.75). The sampling adequacy for factor analysis was proven by a Kaiser–Meyer–Olkin value of 0.84. The goodness of fit (GFI) statistics for the model were in the acceptable range (Chi-square/Degree of freedom (CMIN/DF) = 1.38, Goodness-of-Fit Index (GFI) = 0.88, Comparative Fit Index (CFI) = 0.95, Root Mean Square Error of Approximation (RMSEA) = 0.07). **Conclusions:** The FAB-A has demonstrated excellent psychometric qualities for measuring balance in older adults.

## 1. Introduction

The global old population has been consistently growing alongside an upward trend in longevity [1,2]. According to the World Health Organization (WHO), those aged 60 years and older constitute 12% of the global population. The projected growth rate is estimated to be approximately 3% annually, resulting in a 22% increase by 2050 [3,4]. Arab countries have also experienced comparable patterns of population ageing [5,6]. By 2050, the Arab world is expected to witness a substantial rise in the proportion of adults aged 60 and older, reaching 19% of the overall population. This signifies a considerable increase from the average of almost 7% recorded in 2015 [7,8]. These statistics highlight the imperative to address the health issues associated with ageing in the older population.

Falls are a prevalent health issue among older adults [9,10]. Each year, over 25% of adults aged sixty-five and older experience falls, with this figure increasing with the frequency of falls and age [11]. Falls among the elderly can lead to grave, sometimes lethal, harm such as fractures, joint dislocations, and traumatic brain injuries [12]. Nevertheless, even in the absence of physical harm, a fall can still substantially impact one’s mental well-being, manifesting as a dread of falling that subsequently results in a restriction of participation in everyday activities [13,14]. Falls originate from several causes and can be initiated by complex interactions between various risk factors. These risk factors can be classified into two groups: (i) intrinsic factors which are associated with an individual’s functional and health conditions, such as impaired walking, balance problems, coexisting medical conditions, and cognitive impairments. (ii) Extrinsic risk factors include drugs, environmental dangers, and unsuitable assistive devices [9,11,15]. Prompt identification of older adults at risk of falls is essential for the successful implementation of fall prevention strategies. The main objective of fall prevention in the elderly population is to enhance balance and postural control [16].

Several evaluation techniques and clinical tests have been developed to identify and detect balance performance and gait impairment [17,18]. These include the Timed Up and Go (TUG), Performance-Oriented Mobility Evaluation (POMA), Dynamic Gait Index (DGI), and Berg Balance Scale (BBS) [19,20]. Although research on these measures has shown their utility and reliability in assessing fall risk in older individuals, they have some limitations. The BBS is a highly utilized clinical assessment tool that quantifies balance in the elderly population [21]. Nevertheless, the utilization of the BBS for high-functioning older adults is subject to specific constraints [21]. The BBS authors have identified a shortcoming of the scale, which is the absence of an item that assesses the postural reaction to an external stimulus, also known as reactive postural control. Furthermore, the BBS has demonstrated a ceiling effect when employed to evaluate older persons who are functionally independent, as the items lack sufficient difficulty to identify subtle alterations in balance [21]. The presence of these restrictions underscores the necessity of creating a dependable clinical examination that can detect subtle alterations in balance and is suitable for assessing the capabilities of older adults with advanced functional abilities. In response to these concerns, Debra and her colleagues created the Fullerton Advanced Balance scale (FAB), a 10-item assessment tool that evaluates different aspects of balance through performance-based measures [22,23]. The FAB questions were designed to evaluate several dimensions of balance, including sensory, musculoskeletal, and neuromuscular dimensions [23]. These characteristics collectively affect the capacity of high-performing older adults to maintain balance. The tasks conducted in the FAB assess the subjects’ ability to maintain balance by simulating the balancing movements encountered in daily activities, both static and dynamic in nature [23,24]. These tasks have been demonstrated to be more efficient than their BBS equivalents, requiring 10–12 min to administer compared to the BBS’ 15–20 min. Additionally, they are less susceptible to ceiling effects when evaluating older adults with greater cognitive ability. The FAB has been translated into several languages, including Persian, Turkish, and Korean [25,26,27]. However, no attempts have been made to translate this scale into Arabic. This study aimed to translate the FAB questionnaire into Arabic and assess its validity among a sample of Arabic-speaking older adults.

## 2. Materials and Methods

### 2.1. Sample

This study included 68 older adults residing in the neighborhood. Participants were selected through convenience sampling from diverse Saudi communities and contexts, such as community centers, social gatherings, and mosques (places of worship). Informed consent was obtained from each participant prior to enrolment in the study.

Participants were included in the study if they were aged (≥60 years), had ambulation ability (with or without assistive equipment), and could read and respond to questions. Participants were excluded if they had any orthopaedic conditions that would limit mobility, neurological disorders, cognitive impairments, or severe auditory or visual impairments that could significantly affect balance.

### 2.2. Translation

Debra Rose, the first creator of the scale, granted permission for the translation and adaptation of the FAB into Arabic. To assess the accuracy and quality of the Arabic version of the FAB-A [28], a meticulous multiphase symmetrical translation method was employed during the adaptation procedure. First, two proficient Arab translators, skilled in English, translated the FAB into Arabic. The translators came from diverse backgrounds. One was a physical therapist with expertise in medical terminology, while the other was an independent translator with a strong command of colloquial Arabic. Two Arabic translations of the FAB were created, with each translator producing a single version. Subsequently, a tertiary autonomous translator critically assessed the two versions, comparing between them and with the original document. The three translators stated earlier, who were also part of the study team, adopted a committee-based approach to thoroughly examine the translations and resolve any uncertainties or inconsistencies in the items. A consensus was reached, resulting in the creation of an initial Arabic version of the FAB. Subsequently two English speakers conversant in Arabic, back-translated this version into two English versions. The two translators had comparable qualifications to the initial two translators and were entirely unaware of the original FAB rendition. A multidisciplinary group consisting of translators and research scientists was established to evaluate whether there was semantic and conceptual similarity between the two back translations and the original English FAB and to make any required adjustments. The penultimate iteration of the Arabic FAB was derived in this stage. Face validity refers to the degree to which individuals comprehensively grasp and interpret the information presented in a test for a specific objective [29]. A pilot test of the pre-final version of the Arabic FAB was undertaken with 10 members of the target population to assess the face validity of the instrument (FVI) [30,31]. The participants were instructed to evaluate the clarity and thoroughness of the instructions and items in the instrument using a 4-point Likert scale on a response-process validity questionnaire. Content validity pertains to the degree to which a measurement effectively and comprehensively captures all facets of the notion that it is designed to evaluate [30]. To determine the content validity ratio (CVI) of the instrument, a panel of 10 experts in the field (senior occupational and physical therapists) evaluated the instructions, answer format, and scale items to confirm their conceptual and semantic similarities. During the testing process, the testers were directed to assess each item using a 4-point scale: 1 = not relevant, 2 = somewhat relevant, 3 = quite relevant, 4 = highly relevant. During this phase, all discrepancies or ambiguities were addressed, leading to the creation of the final Arabic version of the FAB. This was prepared for clinical administration to conduct comprehensive psychometric testing on the target population.

### 2.3. Outcome Measures

#### 2.3.1. Fullerton Advanced Balance Scale (FAB)

The FAB is a performance-oriented measure used to evaluate postural control in high-functioning older adults [23]. The assessment comprises 10 items that gauge the capacity to maintain balance while executing a range of dynamic and static tasks. The clinician evaluates each activity using a 5-point Likert scale, with scores ranging from 0 to 4. The overall FAB score can vary from 0 to 40, with higher values suggesting better balance. The FAB requires minimal space and equipment and is relatively quick to administer (10–12 min). Items needed to administer the test were as the follows: a stopwatch, pencil, two rulers (2 and 36 inches), masking tape, a 6-inch bench, and a metronome. The 10-item FAB involves the participant standing with their feet together and eyes closed (item 1), reaching forward to retrieve an object (item 2), turning in a circle (item 3), stepping up and over a bench (item 4), tandem walking (item 5), standing on one leg (item 6), standing on foam with eyes closed (item 7), jumping for distance (item 8), walking with head turns (item 9), and recovering from an unexpected loss of balance (item 10). The FAB has shown high test–retest reliability (0.96) as well as intra- (0.92–1.00) and inter-rater reliability (0.91–0.95) [23,24,32]

#### 2.3.2. Berg Balance Scale (BBS)

The BBS is a 14-item scale that evaluates an individual’s ability to maintain balance while performing various tasks. The items were assessed using an ordinal scale ranging from 0 to 4. A value of 0 indicates the minimal level of functionality, implying dependence or inability to perform the activity. A grade of 4 signifies the utmost level of functionality, denoting complete independence in execution of the job. The individual scores for each task are aggregated to obtain the total score of the BBS, with a maximum possible score of 56. BBS scores ranging from 41 to 56 signify strong balance, scores from 40 to 21 denote adequate balance, and scores from 20 to 0 indicate balance impairment [33,34].

#### 2.3.3. Timed up and Go (TUG)

The TUG is a quantitative evaluation that assesses an individual’s capacity for movement and task completion in terms of functional mobility. During the assessment, participants stood from a sitting position, traversed a distance of 3 m at a normal pace, pivoted at reaching the 3 m mark, return to the starting point, and then resumed a seated posture [35,36].

#### 2.3.4. Falls Efficacy Scale-International (FES-I)

The FES-I comprises 16 items, including the original 10 items from the FES, along with an additional six challenging items. Each activity is rated on a 4-point scale, ranging from 1 (not concerned at all) to 4 (extremely concerned). The final score ranges from 16 (indicating little fear of falling) to 64 (indicating serious concern about falling) [37,38].

### 2.4. Statistical Analysis

Data analysis was conducted using the statistical software SPSS version 23 (SPSS, Inc., Chicago, IL, USA) and Graph Pad Prism version 8.0 (GraphPad Software Inc., La Jolla, CA, USA).

#### 2.4.1. Face Validity and Content Validity

Face validity refers to how well the target audience perceives the content of a test instrument in relation to the test’s setting. Face validity was evaluated using the Face Validity Index (FVI) for items (I-FVI) and the total scale (S-FVI). Values ≥ 0.80 indicated acceptable face validity for both the I-FVI and S-FVI. Content validity refers to the extent to which a measurement effectively and properly covers all dimensions of the notion that it is designed to assess. Content validity was evaluated using the Content Validity Index (CVI) for both items (I-CVI) and the total scale (S-CVI). Values ≥ 0.80 indicated acceptable content validity for S-CVI and ≥0.90 for I-CVI [29,31].

#### 2.4.2. Inter-Rater and Intra-Rater Reliability

Inter-rater reliability assesses the degree of agreement between two evaluators who assess the same subject. Test–retest reliability, or intra-rater reliability, assesses the consistency and stability of the results generated by an instrument over time. Further examination of the Fullerton Advanced Balance Scale was performed within 7–10 days after the initial assessment. The interclass correlation coefficient (ICC) was used to assess the degree of concordance among repeated measurements. ICC estimates and their 95% confidence intervals were calculated using the SPSS statistical package version 23 (SPSS Inc, Chicago, IL, USA) based on a mean-rating (k = 2), with a 2-way random-effects model with consistency in selection. The intraclass correlation coefficient (ICC) was interpreted using the following general guidelines for “normal” or acceptable values: ICC < 0.5: Poor reliability, ICC between 0.5 and 0.75: Moderate reliability, ICC between 0.75 and 0.9: Good reliability and ICC > 0.9: Excellent reliability [39]

#### 2.4.3. Internal Consistency

Internal consistency was used to examine the correlations between the different items on the same instrument. This validates whether the items designed to measure the suggested concept truly generate comparable scores. The internal consistency of the scale items was evaluated by using Cronbach’s alpha coefficient [40]. The accepted reference values for interpreting Cronbach’s alpha are as follows: α ≥ 0.9 indicates excellent reliability; 0.8 ≤ α < 0.9 signifies good reliability; 0.7 ≤ α < 0.8 denotes acceptable reliability; 0.6 ≤ α < 0.7 suggests questionable reliability; 0.5 ≤ α < 0.6 reflects poor reliability; and α < 0.5 represents unacceptable reliability [41].

#### 2.4.4. Concurrent Criterion Validity

This validity assessment concerns the extent to which the results of a certain construct, as measured by an instrument, correspond with the results of existing instruments that evaluate similar constructs. The participants were directed to complete four distinct assessments. (FAB, BBS, TUG, FES-I). The relationships between the various outcome indicators were determined using Spearman’s correlation coefficient [42]. The strength of the correlation was categorized as follows: 0.00 to 0.19 indicates a very weak or negligible correlation; 0.20 to 0.39 signifies a weak correlation; 0.40 to 0.59 represents a moderate correlation; 0.60 to 0.79 denotes a strong correlation; and 0.80 to 1.00 reflects a very strong correlation [43].

#### 2.4.5. Predictive Criterion Validity

Predictive validity refers to an instrument’s capacity to predict future outcomes. Sensitivity, specificity, and cut-off points were determined using the Receiver Operating Curve analysis (ROC) against fallers and non-fallers [24].

#### 2.4.6. Construct Validity

Exploratory factor analysis (EFA) was employed to investigate the factor structure of the complete sample, consisting of (n = 68; EFA) participants. The identification of significant factors was based on eigenvalues exceeding one and a visual evaluation of the scree plot. The structure of the two-factor model, which includes the static and dynamic supporting surface constructs, was verified using confirmatory factor analysis with the AMOS program. This study was undertaken to confirm the relationship between FAB-A items and the two constructs. The analysis generated a CFA model that illustrated the possible correlations among the variables. The model fit was assessed using several statistical measures, including the χ^2^ test, comparative fit index (CFI), chi-square fit statistics/degree of freedom (CMIN/DF), goodness-of-fit index (GFI), and root mean square error of approximation (RMSEA). The indices were used for model comparison, and any models that exhibited inadequate fit or values below acceptable thresholds were adjusted using a modification index [44,45].

### 2.5. Ethics

Participants provided informed consent before enrolment in the study, and ethical approval was secured by the Centre of Excellence in Genomic Medical Research (03-CEGMR-Bioeth-2020) and the National Committee of Bioethics (KACST: HA-02-J-003 on 18 March 2020).

## 3. Results

### 3.1. Cross-Cultural Adaptation

The original FAB consisted of 10 components, which remained unchanged without any significant modifications to their content. The unit of measurement for item 4 was changed from inches to centimeters to comply with the European measurement standards. The instrument’s face validity was determined to be satisfactory using the response process validation approach, with unanimous agreement from 15 raters. The item face validity index (I-FVI) was 0.91, and the scale face validity index (S-FVI) was 0.92. The content validity index (CVI) for both the scale (S-CVI) and individual items (I-CVI) was 0.94.

### 3.2. Participants

A post hoc power analysis was conducted using G*power software (version 3.1.9.7, Dusseldorf, Germany) to assess the adequacy of the sample size (n = 68) with a significance level of α = 0.05. Effect sizes were calculated for three different scales employed to evaluate concurrent criterion validity: BBS (0.75), TUG (0.61), and FES-I (0.52). The statistical power of the sample ranged from 99% to 100% for the three scales.

Table 1 presents the participant’s characteristics. A total of 68 participants aged between 60 and 85 years were included in the study. Fourteen (21%) participants reported experiencing a fall in the last year, and only seven (10%) used walking aids during ambulation.

### 3.3. Reliability

#### Test–Retest Reliability and Internal Consistency

Table 2 displays the descriptive attributes of the FAB items, including the mean, standard deviation, skewness, kurtosis, item-total adjustment, and alpha value if the item was excluded from the scale. The mean score for the Arabic version of the FAB was 30.51 ± 8.04. The revised item-total correlation for the Arabic version of the FAB exceeded 0.30, which satisfies the acceptable standard for scale development. Intraclass Correlation Coefficient (ICC) for the inter-rater tests was calculated as (ICC = 0.96, 95% CI 0.94–0.98, *p* ≤ 0.001), and the intra-rater was (ICC = 0.95, 95% CI 0.92–0.97, *p* ≤ 0.001). The 10-item FAB-A had an internal consistency score (Cronbach’s alpha) of (0.86) indicating a high level of internal consistency.

**Figure 1:**
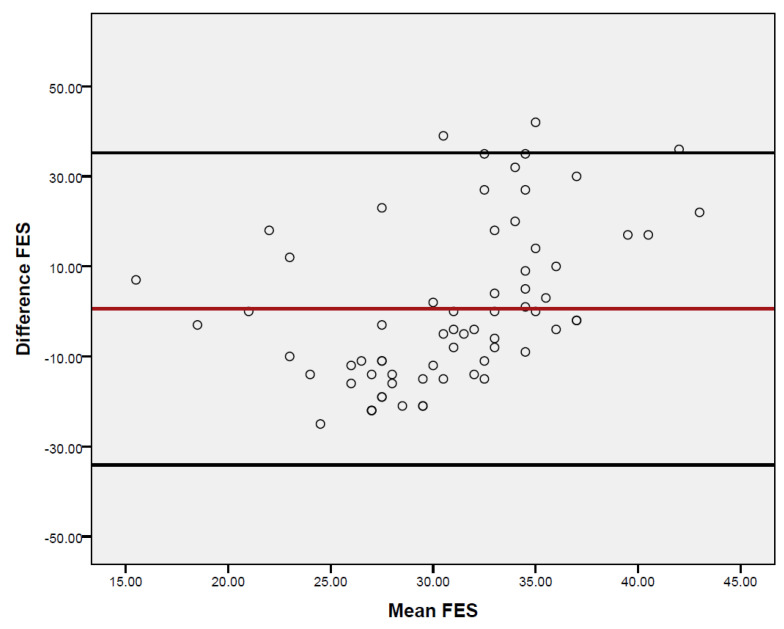
Bland–Altman Plot illustrating the measurements of the FAB-A and FES scales.

Figure 1 displays the Bland–Altman Plot illustrating the measurements of the FAB-A and FES scales, which demonstrates a strong level of agreement. Approximately 96% of the data points fell within a range of ±1.96 standard deviations from the mean difference with (*p* ≤ 0.001).

### 3.4. Validity

#### 3.4.1. Concurrent Criterion Validity

FAB-A showed a statistically significant association compared to BBS, FES-I, and TUG. A strong positive correlation was observed between the mean FAB-A and BBS scores (r = 0.75, *p* ≤ 0.001), whereas a moderate negative association was observed with the FES-I (r = −0.52, *p* ≤ 0.001) and a strong correlation with the TUG (r = −0.61, *p* ≤ 0.001).

#### 3.4.2. Predictive Criterion Validity

**Figure 2:**
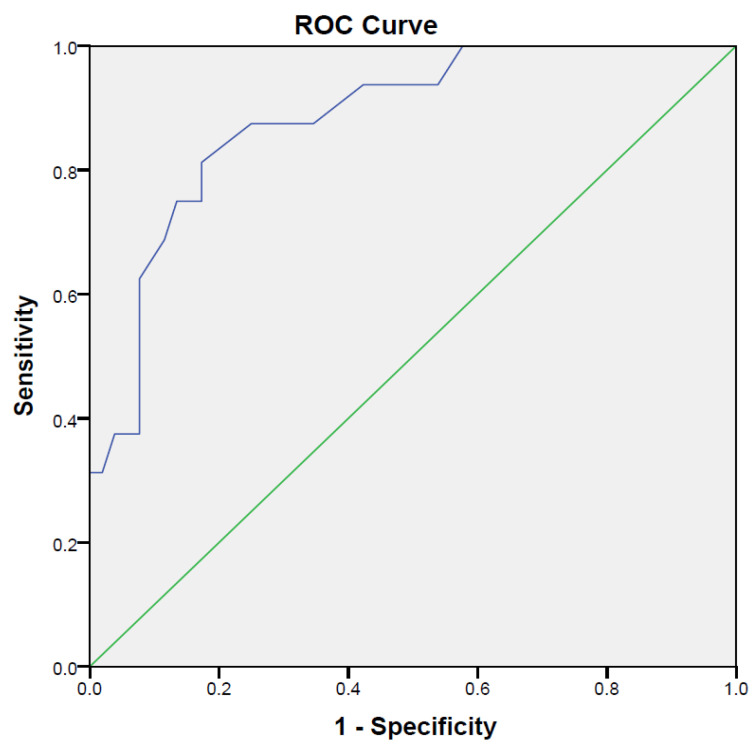
ROC curve illustrating the sensitivity and 1-specificity of FAB-A with fallers and non-fallers.

To determine the predictive validity of FAB-A, an ROC curve was plotted, which determined a cutoff score of 30.5 for FAB-A with an area under the curve of (0.88, *p* ≤ 0.001; 95% CI, 0.79 to 0.97) with a sensitivity of 81.3% and specificity of 82.7%. An additional binary logistic regression analysis was conducted to determine the predictability of FAB-A for those who had falls. The study demonstrated a 79.4% accuracy in predicting fallers, with an Odds Ratio of 3.81 (*p* = 0.032; 95% CI, 1.12 to 12.92), specifically for the fallers group (Figure 2).

#### 3.4.3. Construct Validity

First, preliminary investigations were conducted to determine whether the parametric assumptions of normality were upheld. The Kaiser-Meyer-Olkin (KMO) measure, which evaluates the sufficiency of the sample, produced a result of 0.836. In addition, Bartlett’s test of sphericity yielded a significant result (χ^2^ = 274.096, *p* < 0.001), indicating that the subjects included in the study were appropriate for the data. The scree plot and eigenvalue analysis indicated that the two-factor solution was viable.

**Figure 3:**
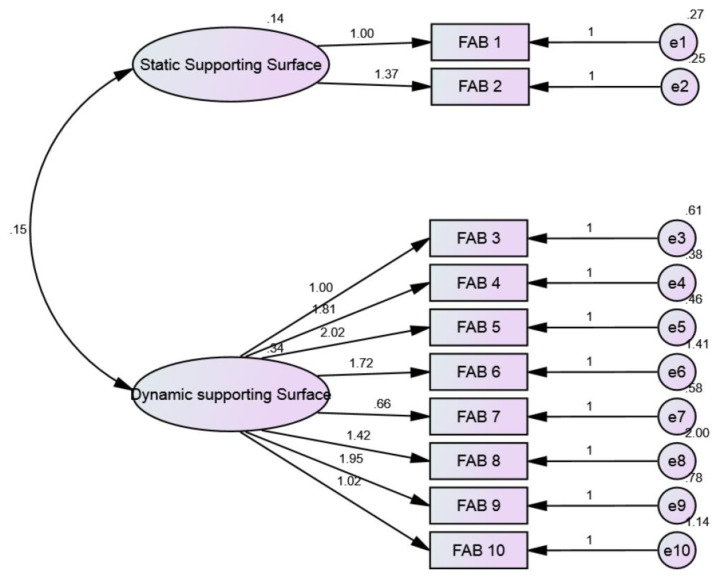
CFA path diagram for the construct of each item of FAB-A.

The exploratory factor analysis yielded a two-factor solution, with the first factor having an eigenvalue of 4.55 (static supporting surface), an eigenvalue of 1.13 for the second factor (dynamic supporting surface), and less than 1 for the other factors. The first and second factors constituted 45.55% and 11.29% of the total sample variance, respectively. These findings indicate that the data were suitable for confirmatory factor analysis. Validation of an acceptable alteration to the two-factor model was accomplished by obtaining statistically significant goodness-of-fit metrics (CMIN/DF = 1.38, GFI = 0.88, CFI = 0.95, RMSEA = 0.07). Figure 3.

## 4. Discussion

Balance issues are a significant constraint on the functioning of elderly individuals. The FAB was designed to assess the functional balance of older adults who are particularly susceptible to falls, and to identify those at a higher risk of falling [23]. The FAB, known for its efficacy in assessing balance proficiency in older adults, has been translated into other languages such as Persian, Turkish, and Korean [25,26,27]. To date, no Arabic version of the scale has been created; hence, this study aimed to translate and verify the FAB within a cohort of Arabic-speaking older adults.

Initially, we translated the items. Subsequently, we determined that no modifications were necessary and carefully replicated each English item’s precise Arabic counterpart. Rose et al. used the systems theory of postural control as a conceptual framework to validate the content of the first edition of the FAB. According to this theory, the musculoskeletal system, sensory and motor neurons, and other systems interact to regulate posture and balance [23]. The systems encompassed in this context are the sensory and musculoskeletal systems, sensory strategies, neuromuscular synergy, internal representations (cognition), and adaptive and anticipatory processes [23]. The FAB-A also demonstrated satisfactory results for both the content and face validity indices, indicating that the instrument is conceptually sound and suitable for conducting comprehensive psychometric testing.

The FAB-A exhibited high internal consistency, as shown by a Cronbach’s alpha coefficient of 0.97. This level of consistency is comparable to other versions of the FAB, including the original form (with H coefficients > 0.75), Korean version (with a Cronbach’s α = 0.92), and Persian version (Cronbach’s α = 0.84) [23,46,47]. The remarkable internal consistency can be ascribed to several factors. The authors of the original FAB study noted that the presence of expert raters, who possess knowledge in evaluating older persons using balance scales, could have influenced the internal consistency results. Moreover, the testing was carried out on a very small sample of older adults who were in a state of good health [23].

The interrater and intrarater ICC values were 0.96 and 0.95, respectively, in our study. The obtained result is similar to the ICC scores of several versions of the FAB, including the original FAB’s inter-rater (0.94–0.97) and intra-rater (0.97–1.00) scores, the Turkish version (inter-rater ICC = 0.92, intra-rater ICC = 0.93), and the German version (ICC = 0.96) [23,25,48]. Inter-rater reliability can be affected by several factors, such as the rater’s level of education, the tiredness levels of both the rater and the responder, and the unique characteristics of the scale being utilized [49]. To counteract the impact of fatigue in our study, the participants completed the tasks for the second assessment after sufficient rest. The raters were provided with task instructions written on boxes to guarantee that the tasks could be easily administered regardless of the examiner’s degree of experience or education. These precautions, in conjunction with previous findings, establish the replicability of the FAB-A results across various instances and with diverse assessors among older persons residing in the Arab community.

In the current study, FAB and BBS presented a strong correlation coefficient of 0.75, which is identical to the original FAB correlation with BBS and similar to the Turkish one (rho = 0.70) [25]. Rose et al. indicated that such a correlation coefficient value indicates similarity between the construct of the two scales however the correlation is not high enough to imply that the two instruments are measuring the same dimensions of balance [23]. FAB-A exhibited a strong negative correlation with the Timed Up and Go (TUG) test (r = −0.61), indicating that higher FAB-A scores correspond to better functional mobility. This finding is consistent with the results reported for the German and Persian versions of the FAB, which demonstrated similar negative correlations of −0.63 and −0.77. Collectively, these results reinforce the validity of the FAB-A as a reliable instrument for assessing balance and mobility, demonstrating alignment with the established international versions [46,48].

Among older adults, falls are a prominent source of injury and illness [10,50]. An extensive evaluation of the likelihood of falling is crucial for reducing the frequency of falls among older adults [51]. The evaluation of fall likelihood involves assessing balance skills, which have been widely studied and proven to be key indicators of fall occurrence [52]. During prospective research evaluating predictive validity, the FAB demonstrated a modest ability to differentiate between those who experienced falls and those who did not, with a cut-off score of (≤27/40) [53]. This cutoff score is similar to the cutoff point estimated by the author of the FAB (≤25/40) [24]. Our study found a strong correlation between the overall score of the scale and the fall occurrence in the previous year. Therefore, by utilizing ROC plots, we established a threshold to distinguish between those who experienced falls and those who did not, to assess the predictive accuracy of the FAB-A in anticipating future falls. The optimal trade-off between the sensitivity and specificity of the ROC curve led to a threshold of (≤30.5/40). A study conducted by Azad et al. discovered that a cut-off point of (≤32/40) may be used to distinguish between those who have experienced falls and those who have not [46]. Individuals with an average score below 30.5 on the FAB assessment should be informed about their susceptibility to future falls and recommended to undergo further testing. Exploratory and confirmatory factor analysis of the FAB-A demonstrated that the sample adequately conformed to the two-factor model. The primary components exerting the most significant influence on FAB among older adults were static and dynamic supporting surfaces. These findings are consistent with those of previous studies [22,46].

The relatively small sample size in this study is partly attributable to the nature of the FAB and the BBS, both of which are performance-oriented assessments. Because these scales require active physical participation and task completion, recruiting a larger sample that can fully perform all required tasks, especially among older adults, can be challenging. This performance-based characteristic inherently limits the pool of eligible participants and may contribute to the reduced sample size, which, in turn, affects the generalizability and robustness of the findings. A larger sample would provide more robust data and enhance the external validity of our results. Secondly, some items within the dynamic balance section of the Fullerton Advanced Balance Scale posed challenges for the older participants. These tasks may have been too physically demanding or complex, leading to a reduced number of completed tasks among this group. This limitation not only restricts the representativeness of the sample but also potentially affects the accuracy of the scale’s assessment in reflecting the true balance abilities of the elderly population. Future research should focus on validating the FAB in diverse clinical populations, such as individuals with stroke and multiple sclerosis. These populations often experience distinct balance impairments that may differ in nature and severity from those observed in the general older adult population. Assessing the scale’s reliability, validity, and sensitivity within these groups would provide valuable insights into its broader applicability and utility in clinical practice.

## 5. Conclusions

The Arabic version of the Fullerton Advanced Balance Scale demonstrated strong psychometric properties, confirming its validity and reliability for assessing balance in older adults. Its high internal consistency and test–retest reliability further support its use in clinical and research settings. The scale’s sensitivity to detect balance impairments makes it an effective measure for identifying individuals at risk of falling. Future studies should explore its applicability across diverse Arabic-speaking populations to enhance generalizability. Consequently, it serves as a valuable tool for clinicians and researchers working with Arabic-speaking older adults to accurately assess and monitor balance performance.

## Figures and Tables

**Table 1:** Characteristics of the participants.

Variables (n = 68)	Frequency (%)	
Sex (male/female)	48 (70.6)/20 (29.4)	
Fall (fallers/non fallers)	14 (20.6)/54 (79.4)	
Assistive device (without/with)	61 (89.7)/7 (10.3)	
	**Mean ± SD (Range)**	**Median, IQR (Range)**
Age (y)	66.10 ± 6.17 (60 to 85)	
FAB	30.51 ± 8.03 (11 to 40)	33 (25 to 37)
BBS	48.19 ± 8.27 (21 to 65)	51 (45 to 54)
TUG (sec)	15.54 ± 5.47 (9 to 36)	14 (12 to 18)
FES	31.08 ± 12.08 (12 to 60)	29 (20 to 39)

**Table 2:** Descriptive statistics of Arabic translated version of FAB.

Item	M	SD	sk	ku	r_it_	a_iid_
FAB (a = 0.86)						
1. Stand with feet together and eyes closed	3.76	0.65	−3.11	9.58	0.36	0.86
2. Reach forward to retrieve an object held at shoulder height with outstretched arm	3.65	0.73	−1.96	2.82	0.46	0.85
3. Turn 360° in right and left directions	3.31	0.98	−1.15	0.04	0.58	0.84
4. Step up onto and over a 6-inch bench	3.29	1.23	−1.66	0.29	0.78	0.82
5. Tandem walk	2.97	1.37	−1.12	−0.06	0.75	0.82
6. Stand on one leg	2.01	1.56	−0.05	−1.49	0.63	0.84
7. Stand on foam with eyes closed	3.63	0.86	−2.65	6.74	0.44	0.85
8. Two footed jump	2.04	1.65	−0.07	−1.63	0.50	0.85
9. Walk with head turns	2.66	1.45	−0.71	−0.92	0.74	0.82
10. Reactive postural control	3.18	1.23	−1.52	1.26	0.45	0.85
Total	30.51	8.04	−0.98	−0.16		

Abbreviations: M: Mean; SD: Standard Deviation; sk: Skewness; ku: Kurtosis; r_it_: Corrected item total correlation; a_iid_: Cronbach’s alpha if item deleted.

## Data Availability

The data presented in this study are available on request from the corresponding author. The data are not publicly available due to ethical restrictions.
